# Evaluation of NEON Data to Model Spatio-Temporal Tick Dynamics in Florida

**DOI:** 10.3390/insects10100321

**Published:** 2019-09-27

**Authors:** Geraldine Klarenberg, Samantha M. Wisely

**Affiliations:** Department of Wildlife Ecology and Conservation, University of Florida, Gainesville, FL 32611, USA; wisely@ufl.edu

**Keywords:** ticks, surveillance, abundance, detection, model, Florida, NEON

## Abstract

In 2013, the National Ecological Observatory Network (NEON) started collecting 30-year multi-faceted ecological data at various spatial and temporal scales across the US including ticks. Understanding the abundance and dynamics of disease vectors under changing environmental conditions in the long-term is important to societies, but sustained long-term collection efforts are sparse. Using hard-bodied tick data collected by NEON, the vegetation and atmospheric data and a statistical state-space model, which included a detection probability component, this study estimated the abundance of tick nymphs and adult ticks across a Florida NEON location. It took into account the spatial and temporal variation, and factors affecting detection. Its purpose was to test the applicability of data collected thus far and evaluate tick abundance. The study found an increase in tick abundance at this Florida location, and was able to explain spatial and temporal variability in abundance and detection. This approach shows the potential of NEON data. The NEON data collection is unique in scale, and promises to be of great value to understand tick and disease dynamics across the US. From a public health perspective, the detection probability of vectors can be interpreted as the probability of encountering that vector, making these types of analyses useful for estimating disease risk.

## 1. Introduction

Ecological monitoring and surveillance systems, by definition, require repeated sampling over time to determine if there has been a change in the system. For disease systems, a change can be defined as an introduction of a new pathogen, vector or host species; the loss of species in an epidemiological disease system; or the change in abundance of a key species in the system. Repeated sampling, however, requires institutional commitment of resources, a sense of urgency to answer questions and to take action on a disease system, as well as the political and social will to maintain that surveillance. These types of monitoring and surveillance systems are rare in ecological or epidemiological systems, particularly on large spatial scales. One example of a national human epidemiological surveillance system is BioSense developed by the Centers for Disease Control and Prevention for the detection and control of bioterrorism and other outbreaks of national concern [1].

For the surveillance of environmental phenomena, the National Ecological Observation Network (NEON) is a continental scale monitoring platform funded by the United States National Science Foundation which aims to collect long-term data at large spatial scales over 30 years with the intent to measure, model and predict ecological change across the United States. The data are collected on more than 600 biogeophysical characteristics at each of the 81 field sites across 20 ecoregions, and started in some locations in 2013 and in most locations by 2018. Of special interest to public health specialists and disease ecologists, the population level data are collected on 3 disease vectors: mosquitos, ticks and small mammals.

Tick-borne diseases are an increasingly large component of the public health burden [2]. The lone star tick, *Amblyomma americanum*, is rapidly expanding westward through the Great Plains of North America, while the deer tick, *Ixodes scapularis*, the American dog tick, *Dermacentor variabilis,* and the Gulf Coast tick, *Amblyomma maculatum* are expanding northward [3]. The deer tick populations have increased in density throughout the northeast and upper Midwestern United States with a concomitant increase in human cases of tick-borne disease [4]. The patterns and processes driving the dramatic change in tick populations throughout the US is slowly being elucidated, but efforts are regional and often uncoordinated on a national level. The CDC, recognizing the public health hazard, has called for an increase in nation-wide surveillance to detect new and emerging tick-borne pathogens and monitor trends in established pathogens and their vectors [5].

Standardized, repeated monitoring over time provides a powerful dataset to understand the changes in an ecosystem and to understand the potential drivers of that change. While the CDC calls for county level sampling to understand the occurrence and prevalence of ticks and their pathogens [6], the NEON dataset was designed to understand population and prevalence trends over a long time series at the ecosystem level [7]. These data have the potential to provide a macroecological perspective on the processes that drive vector population and pathogen epidemiological dynamics. Specifically, the NEON sampling design aims to evaluate interannual changes in the mean or maximum tick abundance, and assess the timing and onset of seasonal cycles of sampled populations within a year (Springer et al., 2016). 

Recent developments in state-space modeling to simulate population dynamics have generated occupancy and abundance models that are well-suited to address the objectives of many types of surveillance studies, and the NEON data conform to the input requirements. These models address the issue of imperfect detection explicitly, allowing for more precise and accurate estimates of populations and for the identification of factors that affect detectability. These models have options to include the variables associated with sites, which adds a spatial dimension to the analysis. The latest iteration of these models also includes a temporal component, thus addressing full temporal-spatial dynamics of populations. NEON sampling, in turn, provides data that is suitable for these models: zero counts are recorded alongside positive counts, and sampling is done at the exact same sites throughout the years. In addition, the site variables are recorded with the tick sampling (elevation, land cover class) or are available from other surveys (vegetation structure, cover, diversity, etc.). The variables that could affect detection, such as the temperature, relative humidity and temperature, are also available. The richness of these data sets allows the development of models that provide insight into the temporal and spatial variability of tick populations, and the factors affecting this variability.

The objective of this study was to use the first five years of the data collected by NEON at its Gulf Coast Forest Ecosystem site in the southeastern domain of the network, Ordway Swisher Biological Station (OSBS), to assess the utility of the dataset to address the aims NEON set out to achieve. The OSBS was one of the first observatories in the system to be completed and as such, has one of the longest temporal datasets. Tick-borne diseases have been understudied in the southeastern United States and the dynamics of tick populations are quite different from the dynamics in the northeastern and midwestern United States [8]. Thus, understanding the changes in tick density over time will provide some insights into the epidemiology of tick-borne diseases in this region. 

## 2. Methods

The general strategy that was used to model the tick dynamics at OSBS was to construct an abundance model using a state-space framework. The tick count data was used as the response variable and multiple environmental variables as explanatory covariates. All data were derived from the publicly available NEON database (https://www.nsf.gov/news/special_reports/neon/).

### 2.1. NEON Data

As part of NEON’s implementation strategy, the U.S. was divided in 20 ecoregions [9,10], with Florida largely falling into the Southeast domain. Each of these domains collects standardized data across multiple sites within the domain. The Ordway-Swisher Biological Station (OSBS) is located in North-Central Florida, just outside the town of Melrose in Putnam County. The OSBS covers a little over 38 km^2^, and the sampling in this location started in 2013. The location contains a tower site and distributed sites for the collection of a variety of data. The tower site contains a flux tower that measures physical and chemical properties on a continuous basis, and also collects atmospheric data, e.g., precipitation, temperature, etc. The distributed site measurements include such variables as vegetation structure, mammal or arthropod occurrences per unit effort, and biogeochemical characteristics. There are a number of distributed sites within the tower site and a number of distributed sites throughout the OSBS. This study only used data from the distributed sites outside the tower site. The data downloaded is reflected in Table 1 [11].

**Figure 1:**
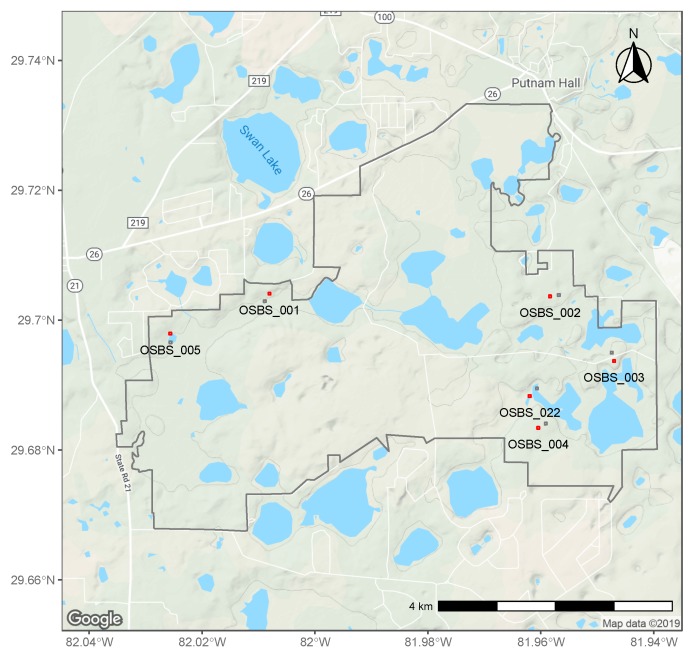
Ordway Swisher Biological Station boundaries with tick sites (red) and distributed sites for vegetation measurements (black).

There were less tick sampling sites at OSBS than sites for vegetation measurements. For this study, only data from distributed sites closest to the tick sites were used (Figure 1). The distance between these sites ranged from 95 m to 123 m. The tick sites and distributed sites were selected in the Ordway Swisher Biological Station area as random stratified samples in such a way that the dominant landcover types (≥5%) were represented: evergreen forest woody wetlands and emergent herbaceous wetlands.

The sampling protocol in this methods section will be described succinctly. More detailed information is available from NEON’s sampling protocols and are cited.

### 2.2. Tick Counts

**Figure 2:**
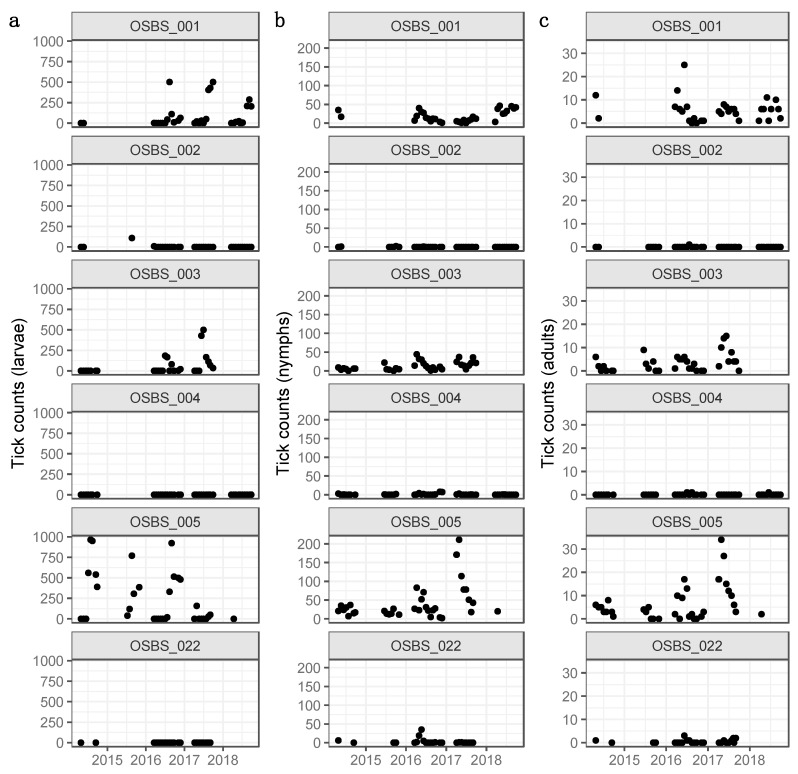
Hard-bodied tick counts at Ordway Swisher Biological Station (Florida) from 2014 to 2018 at each of six sampling sites, separated in (**a**) larvae, (**b**) nymphs and (**c**) adults.

The tick data were collected at 6 sites, which started in 2014 and is ongoing [12]. The tick data from 2014 to 2018 was used for this study. The sampling took place roughly every 3 weeks from approximately March through September/October. Due to the weather conditions and staff availability, data availability was variable between the sites. Site 3 and 22 were flooded after hurricane Irma in September 2017 and have not been accessible since. Each tick site was 40 m by 40 m. The perimeter of the site was sampled with a drag cloth sized 1 × 1 m. If there were shrubs or short trees in the sampling path, these were sampled by flagging. The number of larvae, nymphs and adults was recorded (Figure 2). As the identification of the ticks to species level was not available (analyses are still ongoing), the available counts for hard-bodied ticks as a whole were used. Besides the count data, the sampling method, the sampled area (m^2^), the date of the survey, the time of the survey, the elevation of the sites, the landcover class and the coordinates were recorded, see Supplementary Table S1.

### 2.3. Precipitation

The precipitation data were collected at the NEON tower at 5 min intervals and summed to 30-min data. The daily precipitation values were calculated, but these data were only available for 2017–2018 and contained gaps. The time series were augmented and gap-filled with information from the online National Oceanic and Atmospheric Administration (NOAA) Local Climatological Data tool (https://www.ncdc.noaa.gov/cdo-web/datatools/lcd). The data from the station at Gainesville airport (1645713) were used, which is approximately 29 km from the OSBS. This station provided daily precipitation. A reasonable linear relationship of $P_{OSBS}=1.550+0.599P_{GNVairport}$ with an $R^{2}$ of 0.732 was found (Supplementary Figure S1). From the augmented precipitation time series (spanning 2013 to 2018), this study calculated precipitation on the previous day (of sampling), cumulative precipitation in the previous 7 days and cumulative precipitation in the previous 30 days (Supplementary Figure S4). 

### 2.4. Relative Humidity

The relative humidity was measured at 1-min intervals at the NEON tower and made available as averaged 30-minute data. From these data, the average daily relative humidity was calculated. The relative humidity data were available from 2016 to 2018 and also contained gaps. The daily average humidity data were used from the same NOAA station that also provided the precipitation data to augment and gap-fill the time series. The relationship between the two stations was $RH_{OSBS}=1.036RH_{GNVairport}$ with an $R^{2}$ of 0.997 (Supplementary Figure S2). The augmented time series covered 2013 to 2018 (Supplementary Figure S4).

### 2.5. Temperature

Similar to relative humidity, the temperature was recorded at 1-min intervals and provided as averaged 30-min data. These data were collected at 5 vertical intervals and the data from the lowest level was used. The daily average temperature was calculated and the minimum and maximum temperature for each day was extracted. As with precipitation and relative humidity, the data were limited (2016–2018) and contained gaps. The relationships with data from NOAA (same station) were as follows (Supplementary Figure S3):

$$Tmean_{OSBS}=1.542+0.905Tmean_{GNVairport}\;\left(R^{2}=0.942\right)$$

$$Tmin_{OSBS}=2.187+0.906Tmin_{GNVairport}\;\left(R^{2}=0.923\right)$$

$$Tmax_{OSBS}=0.979+0.975Tmax_{GNVairport}\;\left(R^{2}=0.935\right)$$

The augmented daily time series for these 3 variables covered 2013 to 2019 (Supplementary Figure S4). 

### 2.6. Woody Plant Vegetation Structure

The information on the woody plant vegetation structure contained data from the measurements of live and standing dead woody individuals and shrub groups. A detailed description of the sampling protocol and procedures can be found in various NEON documents [13,14,15]. The distributed sites where the woody plant vegetation structure was measured was 20 m by 20 m. The dataset containing information on apparent individuals was used, which were measurements for each individual with DBH > 10 cm. 

Since not all distributed sites needed for this study were consistently sampled on an annual basis, the average height and stem diameter over all observations for each subplot were calculated as indicators of the woody vegetation structure in the sites (Supplementary Table S2).

### 2.7. Herbaceous Vegetation

Herbaceous clip harvest sampling was conducted on non-woody plants (i.e., grasses, sedges, forbs, bryophytes, non-woody vines), and woody-stemmed plants if their diameter at 10 cm height was < 1 cm. The 20 × 20 m distributed sites contained a randomly located clip-harvest strip of 0.1 m × 2 m, and the aboveground herbaceous biomass was clip-harvested and sorted per functional group. The dry mass (gram) was determined in the laboratory. The dry mass of all functional groups was summed at each site as an indicator of herbaceous vegetation. At the time of our analysis, sampling at the distributed sites that were needed for this study had been conducted once at the OSBS (2013), so these data were not available as a time series (Supplementary Table S2).

### 2.8. Biodiversity and Non-Vegetative Cover

The sites where the plant presence and cover were measured were 20 × 20 m and contained nested subplots of 1 m^2^ and 10 m^2^, (Supplementary Figure S5). At each level, the presence of vascular plants was recorded, their height, and at the 1 m^2^ subplot, the area covered by a particular plant species. For species < 300 cm, the cover included the combined cover of woody and foliar components of the plant, or the herbaceous cover. In the case of plants > 300 cm, the combined cover for the basal diameter, branches and foliage < 300 cm was recorded. Other variables for which cover was estimated were the soil, woody organic material (>5 mm), litter (<5 mm), standing dead material, moss, lichen, feces from wildlife, overstory (until 2015), other non-vascular species (algae, fungus, macrofungi) and other cover (trash, shells). It was possible for the cover types to overlap, so the cumulative cover percentages could add up to more than 100. These data were available on an annual basis for each site. For this analysis, the information on the soil, woody organic material, litter and standing dead material were used as non-vegetative cover data. The overstory was only recorded until 2015, and the other types of cover generally represented < 1% of cover at all sites.

For our calculations, all plants that were identified were used, even if identification was not down to the species level. The species richness (species count) was calculated as well as a biodiversity index (Shannon) at the 1 m^2^ subplot scale. To calculate the Shannon index, the recorded cover percentage was relied on instead of the number of individuals, which was not recorded. The Shannon index calculations based on the percentage cover have been implemented before successfully [16]. To get variables at the site level, the average of the species richness and the Shannon index over the eight 1 m^2^ subplots were calculated. As this sampling was performed annually, these data were compiled for each year for each site. The average non-vegetative cover was calculated across all 1 m^2^ subplots for each site. These data were compiled annually for each site (Supplementary Table S3). 

### 2.9. Abundance Modeling: N-Mixture Models 

The challenge in determining the abundance or population density of a species, is that during surveys, individuals may go undetected. Analyzing the count data without regard of the probability of detection has been shown to lead to biased results [17,18]. The essence of occupancy and abundance modeling is that it specifically accounts for the imperfect detection by including the detection probability. This is similar to classical state-space models that simulate population dynamics. The classical approach models abundance (the true state) as an unmeasured, latent process, and uses observed data conditional on the latent process and an observation error. However, the problems with these classical models are that they make Gaussian assumptions, they assume false positive and false negative observation errors as equally likely, they ignore spatial variation and they assume an equilibrium or no observation error in the first year [19].

The models that were implemented follow the N-mixture modeling approach of Hostetler and Chandler (2015). These models are a form of state-space models as they take process variation and observation error into account, but they resolve the problems of the classical models [19]. The methods build on earlier abundance models and approaches [20,21,22], most notably those by Royle (2004), Kéry et al. (2009) and Dail and Madsen (2011). The model by Royle (2004) first addressed the majority of the issues of the classical models by modeling abundance as an independent random variable according to some distribution (the state of the system), and including the detection probability as a random variable with a binomial distribution (the observation process). Kéry et al. (2009) took this further by addressing the temporal and spatial components. However, this model did not include a component to deal with serial dependence, which is an important feature of state-space models. The model by Dail and Madsen (2011) incorporated a first-order Markov process, and Hostetler and Chandler (2015) extended this model to include the classical population growth formulas and options to deal with zero-inflated data sets. It is important to note that these methods require spatially replicated count data. While the unique identification of individuals is not necessary, the data has to be available from repeat surveys of the same sites as these are required for the estimation of distributions.

The model this study implemented contained three conditionally related processes: (1) initial abundance, (2) abundance at subsequent time periods, which is dependent on the previous time period, and (3) the detection process. The required data are the count data of a species, collected at *R* sites across an area, surveyed during *T* primary sampling periods. Then $X_{i,t}$ is the count data at site *i* (i=1, 2, …,R) at time period *t* (t=1, 2,…,T). The surveys and the detection histories are assumed to be independent of one another. The detection probability (p) is estimated as part of the model to relate the true abundance ($N_{i,t}$) and the count data, if detection probability is perfect, $N_{i,t}\equiv X_{i,t}$. This structure implies that abundance can vary between sites and between primary sampling periods. However, within a primary sampling period, abundance is assumed constant at a site. For each primary sampling period, the model takes all count data for the period at a site and estimates the annual probability distributions for the data. The type of distribution is the same across all sites in an area. The available options are Poisson, zero-inflated Poisson or negative binomial distributions. It starts with the initial year as the first estimate, and the subsequent years take into account the estimate of the previous year, following a first-order Markov process (see Supplementary Methods for details). 

This study tested a number of processes to simulate change, as outlined in Chandler and Hostetler (2015). Abundance can be related to the previous year’s abundance through an exponential function, or by implementing the density-dependent functions, such a Ricker model [23] or a Gompertz-logistic function [24]. Another option is to include a more mechanistic approach, where the abundance is the sum of a survival function and a recruitment function. For the survival function, a survival probability needs to be estimated, for recruitment a recruitment rate, which are both applied to the abundance of the previous year to get abundance for a given year. When taking this approach, there are three options: estimating both these parameters separately to get an autoregressive growth model, making recruitment independent of the previous year’s abundance to get a constant growth model, or setting the recruitment rate as 1 minus the survival rate so the population is in equilibrium (no trend). Thus, in total there were 18 unique combinations (3 distributions types and 6 change functions) available for modeling. The site-level variables can be included in this part of the model, to explain variability between sites. The model simulates the relationships between the initial abundance and site-level variables, and/or the relationships between the parameters in the change functions and site-level variables.

Iteratively, the detection probability was also estimated. While abundance is estimated separately for each site, detection probability is estimated across the whole area (all sites). The detection probability can be dependent on observation-level variables, but these relationships are defined the same across all sites. For instance, if increased humidity decreases the detection probability, the function the model estimates for this relationship is the same across all sites.

The maximum-likelihood estimation (MLE) method was used to estimate the parameters in the model [19,22,25], and the Akaike information criterion (AIC) was calculated to compare candidate models [26,27]. The AIC uses the log-likelihood of the model, but also takes the number estimable parameters of a model into account (Supplementary Methods). It aims to find the most parsimonious model as it tries to balance a good fit and the number of parameters, by essentially penalizing for including too many parameters. The model with the lowest AIC out of a number of candidate models is regarded the best model.

The best model was found for the study area by taking a forward selection approach. This study started by simulating the models with all 18 distribution–change combinations. The distribution and change function of the model with the lowest AIC (the null model) were selected for further modeling. Then, models were developed with variables added one at a time (single variable models), and those where the model AIC was higher than the AIC of the null model were discarded. Of the remaining variables, the models with the observation-level variables only (included in the detection process) and with the site-level variables only (included in the initial abundance determination or change function) were developed. The models were developed with increasingly more variables, the order of which was determined by the AIC of the single variable models. Again, the models with the lowest AIC were deemed the best models. These 2 best models, one for the observation-level variables and one for the site-level variables, were combined into a final model (the full model).

A primary period was defined as a year for our models and included the 6 aforementioned sites. One of the outputs produced by the model was site-specific abundance for each year, which was calculated from the estimated parameters and the actual data with Bayesian inference methods (Supplementary Methods). The model also provided the relationship between the observation-level variables and the detection probability, expressed as linear models with a logit link. The relationships between the site-level variables and initial abundance and/or change were modeled with log-linear models. The abundance of tick nymphs was modeled first and the annual abundance results were used as the site-level variables in the adult model. The efforts to also build a larvae model were unsuccessful due to resource constraints.

For a more extensive overview of the calculations and formulas, see Supplementary Methods, and the original studies that developed these methods [19,22,28].

### 2.10. Software Used, Data and Code Availability

For all data cleaning, analysis and visualization, the R programming language, Version 3.5.3 Great Truth [29] was used. The data were obtained as comma-delimited files from NEON. The package neonUtilities was used for downloading and organizing data. For data cleaning, preparation and visualization, the packages tidyverse, lubridate, lwgeom, psych, vegan, fmsb and cowplot were used. For maps the packages rgdal, sf, ggmap and ggspatial were used. For analyses we used the package unmarked [30,31].

All the data and codes are available from an Open Science Framework repository at https://doi.org/10.17605/OSF.IO/82WN3. 

## 3. Results

Before building the models, a variance inflation factor (VIF) analysis was conducted to eliminate collinear variables (Supplementary Methods). The observation-level variables that remained in the dataset and that were included in the models were the sampling method (drag or drag and flag), the sampled area (m^2^), the average relative humidity, the precipitation of the previous day, the cumulative precipitation of the previous 7 days and previous 30 days, the maximum temperature and the month of the observation and hour of the observation. The latter was rounded to the nearest hour if the observation was not made on the whole hour. The sampling method, month and hour were used in the model as categorical variables. The other observation-level variables were standardized. The once-off site-level variables that remained in the dataset after VIF were the elevation, herbaceous mass and the average height of the woody vegetation. The yearly site-level variables included were species richness, diversity (Shannon index) and the cover of litter, woody organic material, standing dead material and soil. The models for the adult ticks also included the annual nymph abundance estimated with the nymph model.

### 3.1. Distributions, Change Dynamics and Explanatory Variables

A total of 18 combinations of probability distributions and change dynamics were evaluated on their AIC for both nymph and adult models, Table 2. Using negative binomial distributions led to lower AICs for both, but based on a recent study that highlighted the problems with these distributions in abundance models [32] (and see Supplementary Table S4 that shows instability in our case as well), the authors decided to proceed with the next best models. For nymph models, this was a Poisson distribution, for adult models a zero inflated Poisson (ZIP) distribution (shaded in Table 2). For both models, the change dynamics were best modeled with a trend function. i.e. exponential growth or decay. For further modeling, the settings of these null models were used and have been shaded in Table 2.

The results of subsequent models, with the temporal, climatic and environmental variables added to the model one at a time, are reflected in Table 3. The observation-level variables were included in the detection probability component of the model, the once-off site-level variables were used in the initial abundance estimate and the yearly site-level variables in the exponential change function (see Supplementary Methods for details on calculations and equations).

The variables in italics produced the models with higher AICs than the null model and were excluded from further modeling. After adding the variables cumulatively (the order based on the AIC values in Table 3), these models were evaluated again on AIC and the best models with observation-level variables and the best models with site-level variables were selected. These were combined to produce full models (see Supplementary Tables S5 and S6 for an overview and comparison of these models). 

### 3.2. Final Models: Abundance Estimates

**Figure 3:**
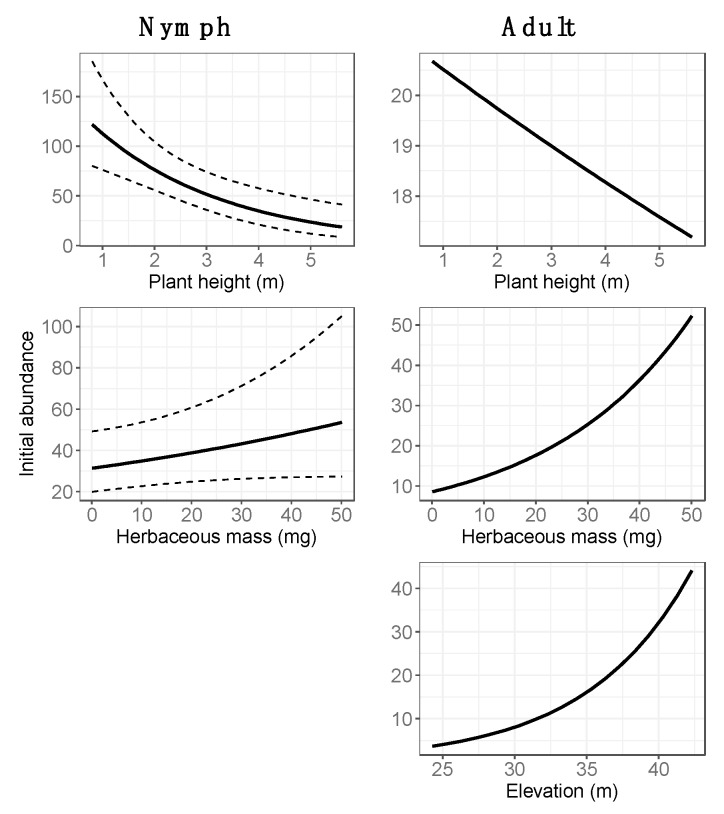
Relationships between initial abundance and site-level variables for nymphs (**left** column) and adult ticks (**right** column), modeled with a log-link function. Dotted lines are the 95% confidence interval. Models for adult ticks have no confidence intervals since the model implementation (package “unmarked” in R) currently does not have a method included to calculate standard errors for ZIP models.

**Figure 4:**
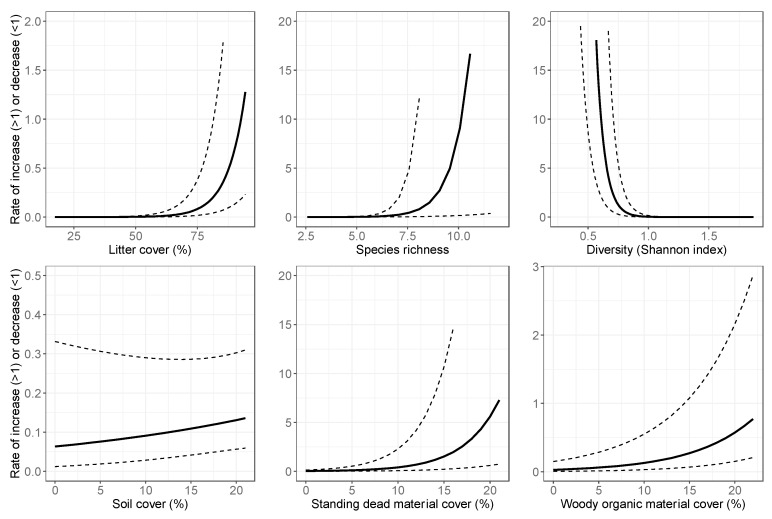
Relationships between yearly site-level variables and population growth rate for nymphs, modeled with a log-link function. Value on the y-axis is what the population from the previous year is multiplied with to get an updated population value. Growth is positive if the value on the y-axis is >1, there is decline if the value is <1. Dotted lines are the 95% confidence interval.

**Figure 5:**
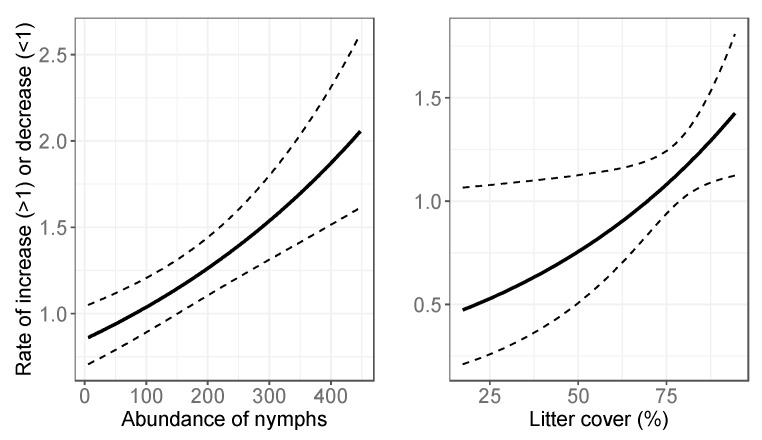
Relationships between yearly site-level variables and population growth rate for adult ticks, modeled with a log-link function. Value on the y-axis is what the population from the previous year is multiplied with to get an updated population value. Growth is positive if the value on the y-axis is >1, there is decline if the value is <1. Dotted lines are the 95% confidence interval.

Based on the model selection procedures, the initial abundance estimates for nymphs were modeled with the average height of woody vegetation and herbaceous mass, and for adults the abundance estimates were modeled with elevation, the average height of woody vegetation and herbaceous mass. The annual growth of nymph abundance was affected by litter cover, species richness, diversity, soil cover, standing dead material cover and woody organic material cover, whereas the annual growth of adult abundance was only affected by the mean nymph abundance and litter cover. Since the site-level variables were not standardized before model development, it was not straightforward to interpret numeric outputs (coefficients) from the models. Instead, Figure 3, Figure 4 and Figure 5 illustrate the relationships between the variables and the initial abundance or growth, modeled with log-link functions. These results show the relationship with each variable separately, while all other variables are held at their average value. The detailed statistical outputs of the models can be found in the Supplementary Tables S7–S10.

**Figure 6:**
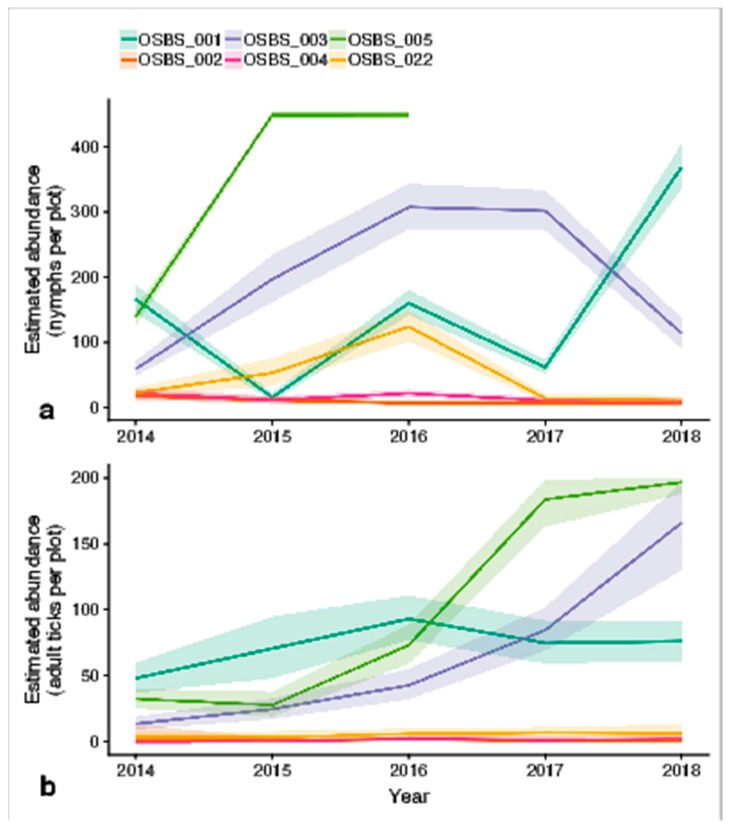
Estimated abundance of (**a**) nymphs and (**b**) adult ticks per site (approx. 160 m^2^). The shaded area is the 95% confidence interval. Site OSBS_005 has no estimates for nymphs for 2017 and 2018 as the model simulated unrealistic exponential growth for these years. Shaded areas are the 95% confidence interval.

The annual abundance estimates after applying the Bayesian inference methods are plotted in Figure 6. The estimates for OSBS_005 were only for 2014, 2015 and 2016. The selected model simulated very large growth rates for 2017 and 2018, causing large population numbers that did not produce estimates with the Bayesian inference methods.

**Figure 7:**
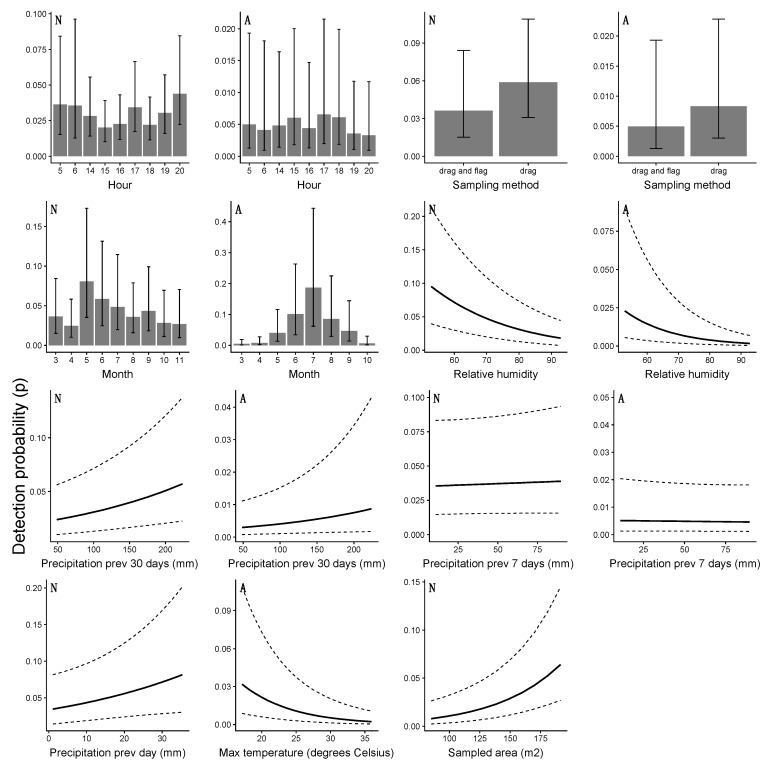
Relationships between observation-level variables and detection probability for nymphs (**N**) and adult ticks (**A**), modeled with a logit link. Dotted lines and whiskers are the 95% confidence interval.

The observation-level variables were standardized before the model development and the coefficients thus indicated the relative importance of the variables (Supplementary Tables S8 and S10). The relationships between the detection probability and the observation-level variables, modeled with a logit link, are visualized in Figure 7. Similar to the site-level variables, these relationships are calculated for each variable separately while all others are kept at their average value.

The model for adults implemented a zero inflated Poisson distribution, and the probability of a Poisson distribution with mean 0 was estimated at 0.33 and thus the probability of a Poisson distribution with a mean other than zero was estimated at 0.67. Both the nymph and the adult tick model included an immigration component (to prevent populations from crashing to zero without recovery), but the estimates were negligible: 0.70 (SE = 1.87) for nymphs and 9.78 (SE = 1.26) for adult ticks (Supplementary Tables S7 and S9).

## 4. Discussion

The models developed in this study estimated abundance on an annual basis, incorporating the detection probability to explain the variability of count data between surveys. The models created probability distributions of abundance for each year at each site, taking the mean as a reflection of abundance for that year. Averaged across all 6 sites over time, our model detected an increase in hard-bodied ticks at the Ordway Swisher Biological Station from 2014–2018. 

### 4.1. Spatial Variation in Abundance

This study included site-level variables to evaluate spatial variability across sites at OSBS. The spatial variability within OSBS was substantial: there were 3 sites with very low abundances for both nymph and adult tick populations. Two sites were classified as evergreen forest, and the other was classified as emergent herbaceous wetlands (OSBS_022). Another site with evergreen forest (OSBS_003) however, did have tick populations. For this reason, more detailed vegetation variables were included in our models than just land cover type to explain this variability. Elevation was also a significant predictor of abundance in our model which was likely correlated with site scale environmental characteristics like soil type or habitat type.

The main differences between the evergeen forest sites with low tick abundance (OSBS_002 and OSBS_004) and the evergreen forest site with ticks (OSBS_003) were lower average vegetation height and lower herbaceous mass at OSBS_003, as well as lower species richness and higher litter cover (Supplementary Table S3). These characteristics highlight the complexity of tick habitats. While lower herbaceous mass is associated with lower initial abundance (Figure 3), it appears that for OSBS_003, the other variables play a strong enough role in supporting tick populations. It was found that instead of land cover classification, finer scale vegetation characteristics are more appropriate for abundance modeling.

Both woody wetland sites (OSBS_001 and OSBS_005) had high tick abundance, but showed different trends. OSBS_001 appeared to have a stable adult tick population, and a variable nymph population, but OSBS_005 showed large increases for both. The nymph populations in the emergent herbaceous wetland (OSBS_022) increased initially but declined after 2016. The evergreen forest site with ticks (OSBS_003) initially increased then decreased in nymph population size, while the adult tick population had an upward trend for the whole period. This could either indicate better than usual survival of nymphs molting into ticks in 2018, or a lag effect that remains unclear for now, since it occurred at the end of the study period. 

To assess what factors played a role in the spatial variability of tick abundance, this study examined the once-off and yearly site-level variables in the models. When the relationships between vegetation structure and nymph and adult abundance were examined, similarities (Figure 3) were found. For both, more herbaceous mass increased initial abundance, and the higher average plant height decreased abundance. The prescribed fire is a regularly applied management tool across the OSBS, and this result affirms that prescribed burning conducted at regular intervals, which reduces herbaceous and understory biomass, is a useful management tool to control tick populations in the Gulf Coast Forest ecosystem, as suggested in other studies [33]. The relationships described by our models are in line with studies that found higher tick densities in areas with a high scrub density (lower average plant height) and an increased density of herbaceous foliage [34]. 

These results are an indication that the model can identify the established relationships reliably, and thus a sign of satisfactory model development. However, the variables associated with variability in initial abundance were limited and lacked complexity, because they were based on a once-off measurement survey at the beginning of the study period. More measurements would have likely provided a better and more nuanced estimate of the relationship between average vegetation height and herbaceous mass and tick abundance. It would have also been desirable to have other site indicators included, such as litter biomass or soil temperature and water content. These variables are only measured in the distributed sites within in the tower site. 

The adult abundance was modeled with a zero inflated Poisson distribution, and the probability of no initial abundance of adult ticks was 0.33. This seems reasonable as 2 out of the 6 sites had low tick counts. Since a regular Poisson distribution was used for nymph abundance, it can be concluded that nymphs were more widespread in OSBS, but that not all sites were favorable for adult ticks. This is most likely associated with site characteristics and host abundance. The hard-bodied ticks likely encountered at OSBS (*Amblyomma americanum*, *Ixodes scapularis*, *A. maculatum* and *Dermacentor variabilis*) have a 3-host life cycle, with nymphs obtaining blood meals from smaller mammals and adults from larger mammals. Large mammals are not part of NEON’s data collection efforts. It is reasonable to suggest that adult ticks would be more abundant in areas where frequent encounters with common large mammals like white-tailed deer (*Odocoileus virginianus*) and wild pigs (*Sus scrofa*) occur. The count data on small mammals are collected by NEON [35,36], but currently not every tick site has a corresponding small mammal trapping site. The missing data eliminates the observations or sites from the model, and this would remove 3 of the 6 sites. While both tick and small mammal trapping sites represent all landcover types and could theoretically be associated with each other based on this information, this proved not to be a good solution. The current tick models and an inspection of the small mammal data showed that there was substantial variation of tick abundance and small mammal counts within the same landcover type. In future analyses, it would be worthwhile to develop abundance models for small mammals and use explanatory variables to make rough estimates of mammal abundance in the tick plots. These results could be used as explanatory variables in tick abundance models. This would require an assessment of available NEON data to decide which variables to include in a small mammal model, as the variables included in these tick models might not be relevant in explaining small mammal abundance. In addition, considering NEON monitors a number of species of small mammals, this could be a multi-species model [37,38].

### 4.2. Temporal Variation in Abundance

The annual abundance estimates for nymphs showed more variation over the study period, which is potentially related to a higher sensitivity to yearly site variables. The nymph model contained 6 yearly site variables, while the adult tick model contained only 2 site variables. The adult tick population growth was driven by the number of nymphs and litter cover, implying that other site (habitat) variables were less important. The population growth of nymphs on the other hand showed the relationships with habitat variables, such as litter cover and cover of woody organic material and standing dead material. Note that in the results, the values of >1 indicate population growth, as this is the factor with which the previous year’s population is multiplied. The values of <1 indicated decline (Supplementary Methods). The soil cover and the cover of woody organic material caused a decline in the ticks for our study sites (values < 1 in Figure 4). The litter cover > 70% caused increases in adult tick populations, and > 92% was linked to increases in nymph populations. The standing dead material > 14% was related to increased nymph populations. The standing dead material also included desiccated herbaceous organic material (from the previous year) [39] which could be driving this relationship. The NEON data do not specify whether the material was woody or herbaceous. 

The overall vegetative diversity also influenced the annual abundance estimates of tick populations. A Shannon index < 0.75 and > 8 species per m^2^ implied increased the nymph populations. The low values for the Shannon index indicate a few species, or if there are a reasonable number of species, that they are unevenly distributed (e.g., one species covering most of the area at the expense of other species). The result from our model suggests that more species, but unevenly proportioned, are beneficial for nymph population growth. After examining our data, only a few subplots were found at our sites met these criteria. Higher Shannon indices are more common in the study area. Species richness was low in general (hence the large confidence interval at high species richness), so it was concluded that in most subplots and sites with a few species that were evenly proportioned, the nymph population growth was kept in check. Habitat alterations such as understory removal or burning would thus be a way to reduce the population growth of nymphs, which would subsequently affect the population growth of adult ticks.

### 4.3. Detection Probability and the Risk of Human Exposure

Methodologically, the observation-level variables addressed the unexplained temporal variance, and site-level variables addressed the unexplained spatial variance. In terms of interpretation, this means that the observation-level variables do not address habitat suitability for ticks as interpreted by other studies [40,41,42] but rather the conditions that make ticks more observable. The sampling method used by NEON (dragging and flagging with a cloth) generally targets questing ticks, which roughly resembles the risk for human exposure to medically important ticks found in Florida [7,12,43,44]. With the method implemented in this study, the authors were able to estimate population abundance and the detection probability which is the specific component of the model that represents the human risk of encountering a tick. Including the variables that affect the detection probability then gives an indication of which circumstances increase or decrease the chances of getting bitten. This study included the time of the year (month) as a variable in this process, which highlighted the intra-annual dynamics of this behavior. From a public health perspective, the seasonal data are more relevant than the population dynamics within a year. The results indicate that NEON data and abundance models can be successfully employed to gain insight into inter- and intra-annual dynamics of tick abundance, specifically related to the different life stages and risk of human exposure.

The variation in detection probability was most strongly associated with the time of the year. If all other variables were at their average, the probability of the detection of nymphs was almost 0.1 in May and almost 0.2 for adults in July. Hence, the probability of humans encountering ticks was highest in May (nymphs) and July (adults), and lowest earlier and later in the year. If nymphs need a blood meal in spring time before molting into adults over summer and fall, they are more actively questing and more detectable during that time of the year. Similarly, adults need a blood meal in summer or fall before laying eggs. The type of modeling conducted is useful to indicate exactly when humans are more at risk of encountering ticks and potentially contracting tick-borne diseases. Since NEON is collecting these data across the United States, this holds great potential to map out the risk of tick encounters for public health purposes nationwide. 

The time of the day affected the detection of nymphs somewhat more than adults, with early mornings and the end of the day being higher risk times. This finding was attributed to the nymphs’ sensitivity to desiccation, avoiding warm and sunny times during the day [45,46]. The sampling method played a relatively minor role in detecting adult ticks, though for both nymph and adult ticks, dragging on its own appeared more effective than dragging and flagging. It is suspected that this result was more related to site characteristics than the sampling method. Flagging is usually necessary in areas with shrubs and trees, rather than herbaceous vegetation. This vegetation offers better hiding opportunities (or alternatively more difficult questing options for ticks) than grass-like vegetation [47].

Long-term precipitation positively influenced the detection probability, more so for nymphs than adults. For nymphs, the precipitation of the previous day also increased the detection probability. This was again ascribed to the risk of desiccation for nymphs. Interestingly, the relationship with relative humidity adds some nuance to this narrative. A higher relative humidity actually decreased the detection probability, but was likely associated with higher daytime temperatures and greater evapo-transpiration. Modeling this variable with a quadratic relationship in the future might clarify this relationship. Higher temperatures also decreased the adult tick detection probabilities, with ticks probably retreating into leaf litter and other hiding places to hydrate [46].

The observation-level variables provided some insight into the factors that are important in detectability, but care must be taken in interpreting the results associated with the temperature, relative humidity and precipitation. These were the (mean) daily data from the tower site or NOAA location and did not reflect the actual temperature, relative humidity and precipitation at each tick sampling site at the actual time of sampling–especially in tick habitats such as litter or grass. However, these data indicate general patterns throughout the year and can be interpreted as average conditions across OSBS, the conditions that people can obtain information on when evaluating the exposure risk.

## 5. Conclusions

While NEON’s stated objective is to assess observatory locations, such as the OSBS, as a whole [7], the methods in this study shed light on the variability between sites within the OSBS and the factors that might explain this variability. Using the NEON data to gain insight into the spatial and temporal dynamics of mosquitos, ticks and small mammals looks promising. The main challenge encountered was matching the tick surveillance data and observation-level and site-level data, since missing data disqualified a survey or site from inclusion in the analysis. While the collection of vegetation structure and plant presence data primarily focused on linkages with data from NEON’s Airborne Observation Platform (which produces such products as LiDAR data) [7], this study has shown they also serve a purpose as explanatory variables in abundance models and the authors strongly support continuing or even increasing these data collection efforts. The authors foresee that in the future, remote sensing data of vegetation may be able to be used in lieu of field measurements, once reliable relationships between the vegetation structure and remote sensing data have been established. This would create the opportunity to evaluate larger areas for tick abundance than just small sites, and would be a major achievement by NSF and NEON. The fact that NEON also records whether vegetation is native or non-native would add another important dimension to these analyses. It would allow for an assessment of the influence of invasive species on abundance of disease vectors, and potentially diseases and parasites (the current OSBS dataset did not have enough instances of non-native species recorded to add this dimension to this analysis). 

The relationship between ticks, their environment and habitat is complex [42,46], and the absence of host abundance and dynamics in these current models is a limitation. For future analyses, it would be interesting to develop abundance models for small mammals and examine the relationship with tick abundance. In order for this to be most effective, it would be imperative that all tick sites have a corresponding small mammal trapping site. This way, small mammal abundance could be included as a yearly site variable in tick abundance models. This would be a fruitful endeavor that could shed further light on quantifying the importance and effect of hosts on tick abundance dynamics.

From a public health perspective, conducting these analyses at other NEON sites can provide valuable insights into spatial and temporal tick abundance dynamics nationwide. It would also reveal the differences and similarities where site characteristics are relevant for tick abundance, and which environmental or temporal variables increase the chances of tick encounters across the United States. Aside from ticks, these analyses could and should also be conducted for mosquitos and small mammals, being important disease vectors. These are likely to be time- and resource-intensive undertakings, but the insight that can be gained from these models and the NEON data will be invaluable, at a scale not yet seen before.

## Figures and Tables

**Table 1:** Data products downloaded using the NEON Data Application Programming Interface (API), on 8 March 2019. Note that not all data was actually available for the whole date range requested.

Data Product ID	Product Name	Site ID	Date Range
DP1.10093.001	Tick sampling	OSBS	1 January 2013–31 December 2018
DP1.10098.001	Woody plant vegetation structure	OSBS	1 January 2013–31 December 2018
DP1.10058.001	Plant presence and percent cover	OSBS	1 January 2013–31 December 2018
DP1.10072.001	Small mammal box trapping	OSBS	1 January 2013–31 December 2018
DP1.10023.001	Herbaceous clip harvest	OSBS	1 January 2013–31 December 2018
DP1.00006.001	Precipitation (tower)	OSBS	1 January 2013–31 December 2018
DP1.00098.001	Relative humidity (tower)	OSBS	1 January 2013–31 December 2018
DP1.00002.001	Single aspirated air temperature (tower)	OSBS	1 January 2013–31 December 2018

**Table 2:** Null models for nymph and adult tick abundance (no variables included) to find the best distribution and growth dynamics combination. Distribution and dynamics used in further modeling are shaded. AIC = Akaike Information Criterion, NB = negative binomial, P = Poisson, ZIP = Zero Inflated Poisson.

Nymphs			Adults		
Distribution	Dynamics	AIC	Distribution	Dynamics	AIC
NB	Trend	2832	NB	Trend	909
NB	Autoregressive	2834	NB	Gompertz	910
NB	Gompertz	2870	NB	Autoregressive	911
NB	Equilibrium	2956	ZIP	Trend	928
NB	Constant growth	2958	ZIP	Autoregressive	930
P	Trend	2968	ZIP	Gompertz	930
ZIP	Trend	2970	P	Trend	971
P	Autoregressive	2970	P	Autoregressive	973
ZIP	Ricker	2972	P	Gompertz	973
ZIP	Autoregressive	2972	NB	Constant growth	974
ZIP	Gompertz	2972	NB	Equilibrium	975
P	Gompertz	3015	ZIP	Constant growth	1053
P	Constant growth	3302	ZIP	Equilibrium	1056
ZIP	Constant growth	3304	P	Constant growth	1108
P	Equilibrium	3320	P	Equilibrium	1130
ZIP	Equilibrium	3322	NB	Ricker	1218
NB	Ricker	3390	ZIP	Ricker	1223
P	Ricker	3466	P	Ricker	1242

**Table 3:** Performance of abundance models with variables added one at a time. Variables listed in italics yielded an Akaike Information Criterion (AIC) higher than the null model when included in the modeling. These were not included in further modeling efforts.

Nymphs		Adult	
**Observation-Level Variable**	**AIC**	**Observation-Level Variable**	**AIC**
Month	2611	Month	748
Hour	2850	Maximum temperature	875
Relative humidity	2929	Hour	908
Total sampled area	2937	Precipitation–30 days	911
Sampling method	2949	Precipitation–7 days	922
Precipitation previous day	2959	Relative humidity	928
Precipitation–30 days	2963	*Sampling method*	*928*
Precipitation–7 days	2967	*Total sampled area*	*930*
*Maximum temperature*	*2969*	*Precipitation previous day*	*930*
**Site-Level Variable**	**AIC**	**Site-Level Variable**	**AIC**
Average height woody vegetation *	2853	Nymphs ^#^	865
Litter cover ^#^	2903	Litter cover ^#^	914
Species richness (vegetation) ^#^	2904	Elevation *	914
Diversity (vegetation, Shannon index) ^#^	2919	Average height woody vegetation *	915
Herbaceous mass *	2920	Herbaceous mass *	923
Soil cover	2952	Woody organic material cover ^#^	926
Standing dead material cover ^#^	2955	*Diversity (vegetation, Shannon index) ^#^*	*928*
Woody organic material cover ^#^	2960	*Species richness (vegetation) ^#^*	*929*
Elevation *	2968	*Soil cover ^#^*	*929*
		*Standing dead material cover ^#^*	*930*

^#^ Yearly site variables used in the change function. * Once-off site variables used in the initial abundance estimate.

## Supplementary Materials

The following are available online at https://www.mdpi.com/2075-4450/10/10/321/s1, Supplementary Methods, Figure S1: Comparison of daily precipitation temperature data from NEON and NOAA data, Figure S2: Comparison of daily relative humidity data from NEON and NOAA data, Figure S3: Comparison of daily mean, minimum and maximum temperature data from NEON and NOAA data, Figure S4 Daily observation level variables, extracted from NEON and NOAA data, Figure S5 Vegetation diversity sampling design [39], Table S1: Site characteristics of tick sites in Ordway Swisher Biological Station (Florida), Table S2: Vegetation structure variables at vegetation sampling sites in Ordway Swisher biological Station (Florida), Table S3 Data on vegetation diversity and non-vegetative cover (percentage) per site per year, Table S4 Overview of distribution – change dynamics combinations that were tested for nymph and adult tick data, with different values for K, Table S5 Overview of the nymph model building process, Table S6 Overview of the adult model building process, Table S7 Nymph abundance model specifications and outputs, Table S8 Backtransformed coefficients for the initial abundance, growth and detection probability functions in the nymph model, Table S9 Adult tick abundance model specifications and outputs, Table S10 Backtransformed coefficients for the initial abundance, growth and detection probability functions in the adult tick model.
