# Supplementary material for: Evaluation of NEON Data to Model Spatio-Temporal Tick Dynamics in Florida
Source: Insects. 2019 Sep 27;10(10):321. doi: 10.3390/insects10100321 (PMC6836180; doi:10.3390/insects10100321)
Supplement: Supplementary file 1 [file insects-10-00321-s001.zip › Tick_abundance_SupMat_tables_FINAL_rev.docx]

**Supplementary Tables**

Supplementary Table S1 Site characteristics of tick sites in Ordway Swisher Biological Station (Florida).

| **Site** | **Land cover type** | **Elevation** | **Latitude** | **Longitude** |
| --- | --- | --- | --- | --- |
| OSBS_001 | Woody wetlands | 34.4 | 29.704063 | -82.008061 |
| OSBS_002 | Evergreen forest | 35.8 | 29.703661 | -81.958365 |
| OSBS_003 | Evergreen forest | 38.2 | 29.693693 | -81.94701 |
| OSBS_004 | Evergreen forest | 42.4 | 29.683402 | -81.960449 |
| OSBS_005 | Woody wetlands | 36.0 | 29.697923 | -82.025623 |
| OSBS_022 | Emergent herbaceous wetlands | 24.3 | 29.688309 | -81.961968 |

Supplementary Table S2 Vegetation structure variables at vegetation sampling sites in Ordway Swisher biological Station (Florida).

| **Plot** | **Land cover class** | **Average height of woody vegetation (sd)** | **Average diameter of woody vegetation (sd)** | **Herbaceous mass** |
| --- | --- | --- | --- | --- |
| OSBS_001 | Woody wetlands | 0.8 (-) | 1.1 (-) | 50.22 |
| OSBS_002 | Evergreen forest | 5.68 (6.01) | 12.94 (11.89) | 17.082 |
| OSBS_003 | Evergreen forest | 2.22 (3.31) | 6.40 (11.81) | 0.08 |
| OSBS_004 | Evergreen forest | 4.51 (5.00) | 8.04 (7.71) | 6.07 |
| OSBS_005 | Woody wetlands | 3.43 (4.85) | 7.34 (8.18) | 29.71 |
| OSBS_022 | Emergent herbaceous wetlands | 5.50 (3.78) | 17.25 (2.76) | 24.99 |

Supplementary Table S3 Data on vegetation diversity and non-vegetative cover (percentage) per site per year. Values are averages over 8 subplots of 1 m^2^ each.

| **Plot** | **Year** | **Shannon index** | **Species richness (count)** | **Litter (< 5 mm)** | **Soil** | **Woody organic material (> 5 mm)** | **Standing dead material** |
| --- | --- | --- | --- | --- | --- | --- | --- |
| OSBS_001 | 2014 | 1.50 | 3.625 | 88.375 | 0 | 7 | 0.375 |
|  | 2015 | 1.07 | 4.375 | 79.375 | 9.75 | 8.625 | 1.375 |
|  | 2016 | 1.28 | 4 | 72.125 | 3.625 | 14.25 | 1.875 |
|  | 2017 | 0.98 | 3.75 | 81.25 | 1.0625 | 13.75 | 1.375 |
|  | 2018 | 1.21 | 3.875 | 81.375 | 0.8125 | 22.5 | 1.875 |
|  |  |  |  |  |  |  |  |
| OSBS_002 | 2014 | 2.30 | 10.25 | 48.75 | 21.375 | 3.1875 | 6.4375 |
|  | 2015 | 2.54 | 13.5 | 53.71 | 1.07 | 2 | 1.07 |
|  | 2016 | 2.26 | 12.125 | 62.875 | 0.125 | 13.4375 | 5.25 |
|  | 2017 | 2.59 | 10.625 | 88.125 | 3.8125 | 7.625 | 1.75 |
|  | 2018 | 2.45 | 12.5 | 85.875 | 1.3125 | 3.125 | 14.125 |
|  |  |  |  |  |  |  |  |
| OSBS_003 | 2014 | 1.13 | 3.14 | 88.5 | 0.1875 | 4.5625 | 0 |
|  | 2015 | 1.54 | 3.5 | 94.625 | 0.125 | 3.5625 | 0.0625 |
|  | 2016 | 0.94 | 3.125 | 94 | 0.3125 | 4.9375 | 0.125 |
|  | 2017 | 1.37 | 3 | 91.875 | 0.625 | 7.5 | 0.0625 |
|  | 2018 | 0.73 | 2.875 | 90.5 | 3.0625 | 6.8125 | 0.25 |
|  |  |  |  |  |  |  |  |
| OSBS_004 | 2014 | 2.67 | 8 | 50.75 | 12 | 2.9375 | 0.5625 |
|  | 2015 | 2.06 | 8.75 | 66 | 2.5625 | 1.75 | 0.5625 |
|  | 2016 | 2.27 | 9.625 | 86 | 2.9375 | 2.9375 | 0.75 |
|  | 2017 | 2.30 | 9.5 | 76.125 | 0.1875 | 12.625 | 3.1875 |
|  | 2018 | 2.09 | 8.875 | 71.625 | 0.6875 | 11.375 | 7.125 |
|  |  |  |  |  |  |  |  |
| OSBS_005 | 2014 | 1.49 | 3.33 | 65.875 | 0 | 4.125 | 11.0625 |
|  | 2015 | 1.50 | 3.67 | 72.43 | 0.71 | 2.86 | 5.29 |
|  | 2016 | 1.11 | 3.5 | 82.25 | 0.125 | 6.625 | 8 |
|  | 2017 | 0.92 | 2.57 | 69.75 | 0 | 6.75 | 5.75 |
|  | 2018 | - | - | - | - | - | - |
|  |  |  |  |  |  |  |  |
| OSBS_022 | 2014 | 0.94 | 3.125 | 66.625 | 0.0625 | 0 | 21.0625 |
|  | 2015 | 0.31 | 3.375 | 51.125 | 0.4375 | 0 | 6 |
|  | 2016 | 0.86 | 4.625 | 55.125 | 0.9375 | 0.1875 | 3.6875 |
|  | 2017 | - | - | - | - | - | - |
|  | 2018 | - | - | - | - | - | - |

Supplementary Table S4 Overview of distribution – change dynamics combinations that were tested for nymph and adult tick data, with different values for K. The parameter K is an integer defining the upper bound of the discrete integration, and should be set higher than the maximum observed count and high enough that it does not affect parameter estimates. A previous study [32] found that many models with a negative binomial distribution are unstable, in the sense that increasing K always keeps reducing the AIC (and increasing abundances). These models showed a similar tendency. The best model after those with negative binomial distributions (in italics) were chosen to continue modeling with, with K=450 for nymph models and K=200 for adult models.

| **Nymphs** | | | | | | | | |
| --- | --- | --- | --- | --- | --- | --- | --- | --- |
| **K = 450** | | | **K = 500** | | | **K = 550** | | |
| Distribution | Dynamics | AIC | Distribution | Dynamics | AIC | Distribution | Dynamics | AIC |
| NB | trend | 2832 | NB | trend | 2830 | NB | trend | 2829 |
| NB | autoreg | 2834 | NB | autoreg | 2832 | NB | autoreg | 2831 |
| NB | gompertz | 2870 | NB | gompertz | 2870 | NB | gompertz | 2870 |
| NB | notrend | 2956 | NB | notrend | 2950 | NB | notrend | 2945 |
| NB | constant | 2958 | NB | constant | 2952 | NB | constant | 2947 |
| *P* | *trend* | *2968* | *P* | *trend* | *2968* | *P* | *trend* | *2968* |
| ZIP | trend | 2970 | ZIP | trend | 2970 | ZIP | trend | 2970 |
| P | autoreg | 2970 | P | autoreg | 2970 | P | autoreg | 2970 |
| ZIP | ricker | 2972 | ZIP | ricker | 2972 | ZIP | autoreg | 2972 |
| ZIP | autoreg | 2972 | ZIP | autoreg | 2972 | ZIP | ricker | 2972 |
| ZIP | gompertz | 2972 | ZIP | gompertz | 2972 | ZIP | gompertz | 2972 |
| P | gompertz | 3015 | P | gompertz | 3015 | P | gompertz | 3015 |
| P | constant | 3302 | P | constant | 3302 | P | constant | 3302 |
| ZIP | constant | 3304 | ZIP | constant | 3304 | ZIP | constant | 3304 |
| P | notrend | 3320 | P | notrend | 3320 | P | notrend | 3320 |
| ZIP | notrend | 3322 | ZIP | notrend | 3322 | ZIP | notrend | 3322 |
| NB | ricker | 3390 | NB | ricker | 3390 | NB | ricker | 3390 |
| **Adults** | | | | | | | | |
| **K = 200** | | | **K = 300** | | | **K = 400** | | |
| Distribution | Dynamics | AIC | Distribution | Dynamics | AIC | Distribution | Dynamics | AIC |
| NB | trend | 909 | NB | trend | 904 | NB | trend | 902 |
| NB | gompertz | 910 | NB | gompertz | 906 | NB | autoreg | 904 |
| NB | autoreg | 911 | NB | autoreg | 906 | NB | gompertz | 904 |
| *ZIP* | *trend* | *928* | *ZIP* | *trend* | *927* | *ZIP* | *trend* | *927* |
| ZIP | autoreg | 930 | ZIP | autoreg | 929 | ZIP | autoreg | 929 |
| ZIP | gompertz | 930 | ZIP | gompertz | 929 | ZIP | gompertz | 929 |
| P | trend | 971 | NB | constant | 967 | NB | constant | 964 |
| P | autoreg | 973 | NB | notrend | 968 | NB | notrend | 964 |
| P | gompertz | 973 | P | trend | 971 | P | trend | 971 |
| NB | constant | 974 | P | autoreg | 973 | P | autoreg | 973 |
| NB | notrend | 975 | P | gompertz | 973 | P | gompertz | 973 |
| ZIP | constant | 1053 | ZIP | constant | 1053 | ZIP | constant | 1053 |
| ZIP | notrend | 1056 | ZIP | notrend | 1056 | ZIP | notrend | 1056 |
| P | constant | 1108 | P | constant | 1108 | P | constant | 1108 |
| P | notrend | 1130 | P | notrend | 1130 | P | notrend | 1130 |
| NB | ricker | 1218 | NB | ricker | 1218 | NB | ricker | 1218 |
| ZIP | ricker | 1223 | ZIP | ricker | 1223 | ZIP | ricker | 1223 |
| P | ricker | 1242 | P | ricker | 1242 | P | ricker | 1242 |

Supplementary Table S5 Overview of the nymph model building process. Separate models with increasingly more observation-level variables and site-level variables were developed (order of addition was based on the results in Table 3, variables of the models with the best AIC added first). The two best models were combined into a final model.

| **Nymph model with increasingly more observation-level variables** | | | | |  |
| --- | --- | --- | --- | --- | --- |
| **Distribution** | **Dynamics** | **Variables** | **AIC** | **Akaike weights** | |
| P | trend | ~ month + hour + precip30 + RH + totalSampledArea + samplingMethod + precip + precip7 | 1965 | 0.634 | |
| P | trend | ~ month + hour + precip30 + RH + totalSampledArea + samplingMethod + precip | 1968 | 0.189 | |
| P | trend | ~ month + hour + precip30 + RH + totalSampledArea + samplingMethod | 1968 | 0.177 | |
| P | trend | ~ month + hour + precip30 + RH + totalSampledArea | 2640 | 0.000 | |
| P | trend | ~ month + hour + precip30 + RH | 2660 | 0.000 | |
| P | trend | ~ month + hour + precip30 | 2691 | 0.000 | |
| P | trend | ~ month + hour | 2695 | 0.000 | |
|  | | | | |  |
| **Nymph model with increasingly more site-level variables** | | | | |  |
| **Distribution** | **Dynamics** | **Variables** | **AIC** | **Akaike weights** | |
| P | trend | ~ ave_height + litter + veg_species + veg_diversity + sum_mass + soil + standingDead + wood | 2641 | 0.999 | |
| P | trend | ~ ave_height + litter + veg_species + veg_diversity + sum_mass + soil | 2655 | 0.001 | |
| P | trend | ~ ave_height + litter + veg_species + veg_diversity + sum_mass + soil + standingDead | 2740 | 0.000 | |
| P | trend | ~ ave_height + litter + veg_species + veg_diversity + sum_mass | 2745 | 0.000 | |
| P | trend | ~ ave_height + litter + veg_species + veg_diversity | 2759 | 0.000 | |
| P | trend | ~ ave_height + litter + veg_species | 2766 | 0.000 | |
| P | trend | ~ ave_height + litter | 2801 | 0.000 | |
|  | | | | |  |
| **Final nymph model (site-level + observation level covariates)** | | | | |  |
| **Distribution** | **Dynamics** | **Variables** | **AIC** |  | |
| P | trend | ~ ave_height + litter + veg_species + veg_diversity + sum_mass + soil + standingDead + wood | 2404 |  | |
|  |  | ~ month + hour + precip30 + RH + totalSampledArea + samplingMethod + precip + precip7 |  |  | |

Supplementary Table S6 Overview of the adult model building process. Separate models with increasingly more observation-level variables and site-level variables were developed (order of addition was based on the results in Table 3, variables of the models with the best AIC added first). The two best models were combined into a final model.

| **Adult model with increasingly more observation-level variables** | | | | | |
| --- | --- | --- | --- | --- | --- |
| **Distribution** | **Dynamics** | **Variables** | **AIC** | | **Akaike weights** |
| ZIP | trend | ~ month + maxTemp + hour + precip30 + precip7 + RH | 665 | | 1.000 |
| ZIP | trend | ~ month + maxTemp + hour | 745 | | 0.000 |
| ZIP | trend | ~ month + maxTemp + hour + precip30 + precip7 | 745 | | 0.000 |
| ZIP | trend | ~ month + maxTemp + hour + precip30 | 746 | | 0.000 |
| ZIP | trend | ~ month + maxTemp | 750 | | 0.000 |
| **Adult model with increasingly more site-level variables** | | | | | |
| **Distribution** | **Dynamics** | **Variables** | **AIC** | **Akaike weights** | |
| ZIP | trend | ~ nymphs + litter + elevation + ave_height + sum_mass | 828 | 0.729 | |
| ZIP | trend | ~ nymphs + litter + elevation + ave_height + sum_mass + wood | 830 | 0.268 | |
| ZIP | trend | ~ nymphs + litter + elevation + ave_height | 839 | 0.003 | |
| ZIP | trend | ~ nymphs + litter + elevation | 855 | 0.000 | |
| ZIP | trend | ~ nymphs + litter | 857 | 0.000 | |
| **Final adult model (site-level + observation level covariates)** | | | | | |
| **Distribution** | **Dynamics** | **Variables** | **AIC** |  | |
| ZIP | trend | ~ nymphs + litter + elevation + ave_height + sum_mass | 610 |  | |
|  |  | ~ month + maxTemp + hour + precip30 + precip7 + RH |  |  | |

Supplementary Table S7 Nymph abundance model specifications and outputs

Call:

pcountOpen(lambdaformula = ~ave_height + sum_mass, gammaformula = ~litter +

veg_species + veg_diversity + soil + standingDead + wood,

omegaformula = ~1, pformula = ~month + hour + precip30 +

RH + totalSampledArea + samplingMethod + precip + precip7,

data = occu_file, mixture = "P", K = 450, dynamics = "trend",

immigration = TRUE)

Abundance (log-scale):

Estimate SE z P(>|z|)

(Intercept) 4.8863 0.38245 12.78 2.22e-37

ave_height -0.3908 0.10703 -3.65 2.61e-04

sum_mass 0.0107 0.00667 1.61 1.08e-01

Growth Rate (log-scale):

Estimate SE z P(>|z|)

(Intercept) -8.3403 1.8603 -4.48 7.35e-06

litter 0.1413 0.0143 9.85 6.58e-23

veg_species 1.2115 0.3460 3.50 4.63e-04

veg_diversity -14.6351 1.5423 -9.49 2.33e-21

soil 0.0362 0.0313 1.16 2.48e-01

standingDead 0.2616 0.0409 6.40 1.59e-10

wood 0.1492 0.0184 8.10 5.68e-16

Detection (logit-scale):

Estimate SE z P(>|z|)

(Intercept) -3.2790 0.4558 -7.1943 6.28e-13

month4 -0.3972 0.2184 -1.8188 6.89e-02

month5 0.8436 0.1460 5.7781 7.55e-09

month6 0.5019 0.1980 2.5356 1.12e-02

month7 0.3037 0.2044 1.4856 1.37e-01

month8 -0.0112 0.1841 -0.0610 9.51e-01

month9 0.1867 0.2033 0.9181 3.59e-01

month10 -0.2553 0.2406 -1.0611 2.89e-01

month11 -0.3140 0.2933 -1.0706 2.84e-01

hour6 -0.0197 0.5094 -0.0386 9.69e-01

hour13 -6.0535 9.2805 -0.6523 5.14e-01

hour14 -0.2586 0.3096 -0.8355 4.03e-01

hour15 -0.6062 0.3002 -2.0191 4.35e-02

hour16 -0.4871 0.3114 -1.5643 1.18e-01

hour17 -0.0567 0.3292 -0.1723 8.63e-01

hour18 -0.5158 0.3227 -1.5982 1.10e-01

hour19 -0.1835 0.3135 -0.5853 5.58e-01

hour20 0.1955 0.3425 0.5707 5.68e-01

hour21 -4.0436 3.1222 -1.2951 1.95e-01

precip30 0.2530 0.0747 3.3891 7.01e-04

RH -0.3965 0.0624 -6.3514 2.13e-10

totalSampledArea 0.2146 0.0516 4.1616 3.16e-05

samplingMethoddrag and flag 0.5053 0.2952 1.7118 8.69e-02

precip 0.1227 0.0618 1.9845 4.72e-02

precip7 0.0250 0.0568 0.4397 6.60e-01

Immigration (log-scale):

Estimate SE z P(>|z|)

2.28 0.234 9.76 1.64e-22

AIC: 2404.351

Number of sites: 6

optim convergence code: 0

optim iterations: 288

Bootstrap iterations: 0

Supplementary Table S8 Backtransformed coefficients for the initial abundance, growth and detection probability functions in the nymph model. See Supplementary Methods for more detail on the equations. ** indicates significance at p < 0.05 and * indicates significance at p < 0.10. Observation-level variables used in the detection probability function were standardized. Month, hour and sampling method were included as categorical variables.

| **Variable** | **Coefficient** | **SE** | **p-value** |
| --- | --- | --- | --- |
| **Initial abundance (**$\boldsymbol{\lambda}$**)^1^** | | | |
| Intercept | 132.47 | 1.47 | 0.00** |
| Ave_height | 0.68 | 1.11 | 0.00** |
| Sum_mass | 1.01 | 1.01 | 0.11 |
| **Growth rate (**$\boldsymbol{\gamma}$**)^2^** | | | |
| Intercept | 0.00^#^ | 6.43 | 0.00** |
| Litter | 1.15 | 1.01 | 0.00** |
| Veg_species | 3.36 | 1.41 | 0.00** |
| Veg_diversity | 0.00^##^ | 4.68 | 0.00** |
| Soil | 1.04 | 1.03 | 0.25 |
| StandingDead | 1.30 | 1.04 | 0.00** |
| Wood | 1.16 | 1.02 | 0.00 |
| **Detection probability (p)^3^** | | | |
| Intercept | 0.04 | 0.61 | 0.00** |
| month4 | 0.40 | 0.55 | 0.07* |
| month5 | 0.70 | 0.54 | 0.00** |
| month6 | 0.62 | 0.55 | 0.01** |
| month7 | 0.58 | 0.55 | 0.14 |
| month8 | 0.50 | 0.55 | 0.95 |
| month9 | 0.55 | 0.55 | 0.36 |
| month10 | 0.44 | 0.56 | 0.29 |
| month11 | 0.42 | 0.57 | 0.28 |
| hour6 | 0.50 | 0.62 | 0.97 |
| hour13 | 0.00 | 1.00 | 0.51 |
| hour14 | 0.44 | 0.58 | 0.40 |
| hour15 | 0.35 | 0.57 | 0.04** |
| hour16 | 0.38 | 0.58 | 0.12 |
| hour17 | 0.49 | 0.58 | 0.86 |
| hour18 | 0.37 | 0.58 | 0.11 |
| hour19 | 0.45 | 0.58 | 0.56 |
| hour20 | 0.55 | 0.58 | 0.57 |
| hour21 | 0.02 | 0.96 | 0.20 |
| precip30 | 0.56 | 0.52 | 0.00** |
| RH | 0.40 | 0.52 | 0.00** |
| totalSampledArea | 0.55 | 0.51 | 0.00** |
| samplingMethoddrag and flag | 0.62 | 0.57 | 0.09* |
| precip | 0.53 | 0.52 | 0.05** |
| precip7 | 0.51 | 0.51 | 0.66 |

^1^ $\log\left( \lambda_{i} \right)=\beta_{0}^{\lambda}+\beta_{1}^{\lambda}y_{1,i}+\beta_{1}^{\lambda}y_{2,i}\ldots\beta_{s}^{\lambda}y_{s,i}$ for $N_{i,1} \sim\mathrm{Pois}\left( \lambda_{i} \right)$

^2^ $\log\left( \gamma_{i,t} \right)=\beta_{0}^{\gamma}+\beta_{1}^{\gamma}y_{1,i,t}+\beta_{1}^{\gamma}y_{2,i,t}\ldots\beta_{s}^{\gamma}y_{s,i,t}$ for $\gamma_{i,t}=e^{r_{i,t}}$ in $N_{i,t} \sim\mathrm{Pois}\left( {N_{i,t-1} e}^{r_{i,t}} \right)$

^3^ $\ln\left( \frac{p_{i,j,t}}{1-p_{i,j,t}} \right)=\beta_{0}^{p}+\beta_{1}^{p}z_{1,i,j,t}+\beta_{2}^{p}z_{2,i,j,t}\ldots+\beta_{1}^{p}z_{q,i,j,t}$

With $N$ = abdundance, $i$ = site, $s$ = site-level variable, $t$ = primary period (year), $j$ = survey, $q$ = observation-level covariate.

^#^ 0.00024

^##^ 0.00000044

Supplementary Table S9 Adult tick abundance model specifications and outputs

Call:

pcountOpen(lambdaformula = ~elevation + ave_height + sum_mass,

gammaformula = ~nymphs + litter, omegaformula = ~1, pformula = ~month +

maxTemp + hour + precip30 + precip7 + samplingMethod +

RH, data = occu_file, mixture = "ZIP", K = 200, dynamics = "trend",

immigration = TRUE)

Abundance (log-scale):

Estimate SE z P(>|z|)

(Intercept) -2.2948 3.1189 -0.736 0.46186

elevation 0.1384 0.0746 1.856 0.06349

ave_height -0.0385 0.1489 -0.258 0.79603

sum_mass 0.0362 0.0117 3.080 0.00207

Growth Rate (log-scale):

Estimate SE z P(>|z|)

(Intercept) -1.21617 0.530240 -2.29 2.18e-02

nymphs 0.00197 0.000404 4.87 1.11e-06

litter 0.01432 0.006628 2.16 3.08e-02

Detection (logit-scale):

Estimate SE z P(>|z|)

(Intercept) -5.2987 0.7002 -7.5676 3.80e-14

month4 0.4493 0.4967 0.9045 3.66e-01

month5 2.1309 0.3742 5.6943 1.24e-08

month6 3.1156 0.4742 6.5703 5.02e-11

month7 3.8254 0.4751 8.0525 8.11e-16

month8 2.9276 0.5330 5.4928 3.96e-08

month9 2.2858 0.5786 3.9506 7.79e-05

month10 0.2957 0.5220 0.5665 5.71e-01

month11 -12.2379 153.1546 -0.0799 9.36e-01

maxTemp -0.6646 0.1552 -4.2831 1.84e-05

hour6 -0.1874 0.5616 -0.3337 7.39e-01

hour13 0.2295 1.1872 0.1933 8.47e-01

hour14 -0.0325 0.3832 -0.0847 9.32e-01

hour15 0.1922 0.3563 0.5393 5.90e-01

hour16 -0.1201 0.3730 -0.3219 7.48e-01

hour17 0.2786 0.3528 0.7898 4.30e-01

hour18 0.2073 0.3424 0.6054 5.45e-01

hour19 -0.3325 0.3554 -0.9356 3.49e-01

hour20 -0.4147 0.4065 -1.0202 3.08e-01

hour21 0.1604 0.4391 0.3652 7.15e-01

precip30 0.3015 0.1591 1.8947 5.81e-02

precip7 -0.0287 0.0821 -0.3494 7.27e-01

samplingMethoddrag and flag 0.5192 0.4636 1.1201 2.63e-01

RH -0.5971 0.0843 -7.0792 1.45e-12

Immigration (log-scale):

Estimate SE z P(>|z|)

-0.358 0.625 -0.573 0.567

Zero-inflation (logit-scale):

Estimate SE z P(>|z|)

-0.694 0.867 -0.801 0.423

AIC: 610.0818

Number of sites: 6

optim convergence code: 0

optim iterations: 259

Bootstrap iterations: 0

Supplementary Table S10 Backtransformed coefficients for the initial abundance, growth and detection probability functions in the adult tick model. See Supplementary Methods for more detail on the equations. ** indicates significance at p < 0.05 and * indicates significance at p < 0.10. Observation-level variables used in the detection probability function were standardized. Month, hour and sampling method were included as categorical variables.

| **Variable** | **Coefficient** | **SE** | **p-value** |
| --- | --- | --- | --- |
| **Initial abundance (**$\boldsymbol{\lambda}$**)^1^** | | | |
| Intercept | 0.10 | 22.62 | 0.46 |
| Elevation | 1.15 | 1.08 | 0.06* |
| Ave_height | 0.96 | 1.16 | 0.80 |
| Sum_mass | 1.04 | 1.01 | 0.00** |
| **Growth rate (**$\boldsymbol{\gamma}$**)^2^** | | | |
| Intercept | 0.30 | 1.70 | 0.02** |
| gamTrend(nymphs) | 1.00 | 1.00 | 0.00** |
| gamTrend(litter) | 1.01 | 1.01 | 0.03** |
| **Detection probability (p)^3^** | | | |
| Intercept | 0.00^#^ | 0.67 | 0.00** |
| month4 | 0.61 | 0.62 | 0.37 |
| month5 | 0.89 | 0.59 | 0.00** |
| month6 | 0.96 | 0.62 | 0.00** |
| month7 | 0.98 | 0.62 | 0.00** |
| month8 | 0.95 | 0.63 | 0.00** |
| month9 | 0.91 | 0.64 | 0.00** |
| month10 | 0.57 | 0.63 | 0.57 |
| month11 | 0.00 | 1.00 | 0.94 |
| maxTemp | 0.34 | 0.54 | 0.00** |
| hour6 | 0.45 | 0.64 | 0.74 |
| hour13 | 0.56 | 0.77 | 0.85 |
| hour14 | 0.49 | 0.59 | 0.93 |
| hour15 | 0.55 | 0.59 | 0.59 |
| hour16 | 0.47 | 0.59 | 0.75 |
| hour17 | 0.57 | 0.59 | 0.43 |
| hour18 | 0.55 | 0.58 | 0.54 |
| hour19 | 0.42 | 0.59 | 0.35 |
| hour20 | 0.40 | 0.60 | 0.31 |
| hour21 | 0.54 | 0.61 | 0.71 |
| precip30 | 0.57 | 0.54 | 0.06* |
| precip7 | 0.49 | 0.52 | 0.73 |
| samplingMethoddrag and flag | 0.63 | 0.61 | 0.26 |
| RH | 0.36 | 0.52 | 0.00** |

^1^ $\log\left( \lambda_{i} \right)=\beta_{0}^{\lambda}+\beta_{1}^{\lambda}y_{1,i}+\beta_{1}^{\lambda}y_{2,i}\ldots\beta_{s}^{\lambda}y_{s,i}$ for $N_{i,1} \sim\mathrm{Pois}\left( \lambda_{i} \right)$

^2^ $\log\left( \gamma_{i,t} \right)=\beta_{0}^{\gamma}+\beta_{1}^{\gamma}y_{1,i,t}+\beta_{1}^{\gamma}y_{2,i,t}\ldots\beta_{s}^{\gamma}y_{s,i,t}$ for $\gamma_{i,t}=e^{r_{i,t}}$ in $N_{i,t} \sim\mathrm{Pois}\left( {N_{i,t-1} e}^{r_{i,t}} \right)$

^3^ $\ln\left( \frac{p_{i,j,t}}{1-p_{i,j,t}} \right)=\beta_{0}^{p}+\beta_{1}^{p}z_{1,i,j,t}+\beta_{2}^{p}z_{2,i,j,t}\ldots+\beta_{1}^{p}z_{q,i,j,t}$

With $N$ = abdundance, $i$ = site, $s$ = site-level variable, $t$ = primary period (year), $j$ = survey, $q$ = observation-level covariate.

^#^ 0.00497
