# Supplementary material for: Evaluation of NEON Data to Model Spatio-Temporal Tick Dynamics in Florida
Source: Insects. 2019 Sep 27;10(10):321. doi: 10.3390/insects10100321 (PMC6836180; doi:10.3390/insects10100321)
Supplement: Supplementary file 1 [file insects-10-00321-s001.zip › Tick_abundance_SupMat_methods_FINAL_rev.docx]

**Supplementary Methods**

*Formulas and calculations*

Initial abundance, i.e. abundance in the first primary period, is modeled as a random variable with a particular distribution, e.g.:

$$N_{i,1} \sim\mathrm{Pois}\left( \lambda_{i} \right)$$

where $\lambda_{i}$ is the expected abundance at site $i$ in year 1, in this case a Poisson random variable. The model implementation allows the use of other distribution such as a negative binomial or a zero-inflated Poisson (ZIP) distribution. A ZIP distribution is modeled as

$$N_{i,1} \sim\left\{ \begin{aligned} \mathrm{Pois}\left( 0 \right) with probability \psi\\ \mathrm{Pois}\left( \lambda_{i} \right) with probability \left( 1-\psi\right) \end{aligned} \right.$$

with $\psi$ representing the proportion of zeros. Spatial variability can be accounted for in these models by including $s$ number of site-level variables ($y_{s,i}$) affecting abundance by incorporating a log-linear relationship:

$$\log\left( \lambda_{i} \right)=\beta_{0}^{\lambda}+\beta_{1}^{\lambda}y_{1,i}+\beta_{1}^{\lambda}y_{2,i}\ldots\beta_{s}^{\lambda}y_{s,i}$$

The abundance in the subsequent primary time periods can be modeled in a number of ways, but they have in common that in all of them abundance follows a first-order Markov process: abundance at time $t+1$ is dependent on abundance at time $t$. In brief, the processes that can be incorporated are either composed of a survival and recruitment rate, an exponential growth or decay function, or density-dependent growth functions such as Ricker and Gompertz equations. To allow for environmental variability, all these options are incorporated in such a way that abundance is modeled as a random variable with a certain distribution, as for the initial abundance. We will only briefly outline these processes here, and for further details on these models refer to Dail & Madsen (2011) and Hostetler & Chandler (2015). For ease of reading, we give the formulas using a Poisson distribution, but this could also be a ZIP or negative binomial distribution. All the formulas below are for $t=2,\ldots, T$.

The exponential growth or decay formula for abundance at site $i$ at time period $t$ is

$$N_{i,t} \sim\mathrm{Pois}\left( {N_{i,t-1} e}^{r_{i,t}} \right)$$

where $e^{r_{i,t}}$ is the maximum per capita rate of increase or decrease (also denoted as $\gamma_{i,t}$ in the model).

The density-dependent models involve parameters for the stable equilibrium of the population ($K$) and the instantaneous growth rate at low population densities ($r$), both constrained to be positive. The stochastic version of the Ricker model is

$$N_{i,t} \sim\mathrm{Pois}\left( N_{i,t-1}e^{r\left( 1-\frac{N_{i,t-1}}{K} \right)} \right)$$

Along the same lines the Gompertz model is

$$N_{i,t-1} \sim\mathrm{Pois}\left( N_{i,t-1}e^{r\left( 1-\frac{\log\left( N_{i,t-1}+1 \right)}{\log\left( K+1 \right)} \right)} \right)$$

The process where the population is dependent on survival and recruitment [7] is coined the autoregressive model:

$$S_{i,t}\left| N_{i,t-1} \sim\mathrm{Bin}\left( N_{i,t-1},\omega\right) \right.$$

$$G_{i,t}\left| N_{i,t-1} \sim\mathrm{Pois}\left( \gamma\left( N_{i,t-1} \right) \right) \right.$$

$$N_{i,t} \sim S_{i,t}+G_{i,t}$$

where $S_{i,t}$is the number of individuals surviving (and not emigrating). This is a binomial distribution, since they either survive or not survive, with $\omega$ being the survival probability. Then, $G_{i,t}$is the number of individuals being added to the population, with recruitment rate $\gamma$. The abundance is the sum of the surviving individuals and the individuals added to the population. If we assume the population is at an equilibrium, we need a model without a trend, which is done by adjusting the recruitment rate to

$$\gamma=\left( 1-\omega\right)\lambda$$

From a spatial perspective, while the population is assumed to be at an equilibrium, this still allows us to model animal movement between sites. Finally, if we want to model a scenario where recruitment does not depend on the population in the previous primary period (the “constant” model), we adjust the recruitment function as

$$G_{i,t} \sim\mathrm{Pois}\left( \gamma\right)$$

As in the estimation of the initial abundance, we can include site-level variables to account for variability. The relationships between variables and recruitment rate ($\gamma$ in the autoregressive, equilibrium and constant models), population growth rate ($r$ for the exponential and density dependent models) and the equilibrium abundance ($K$ in the density dependent models) are modeled with a log link. The relationship for survival probability ($\omega$ in the autoregressive, equilibrium and constant models) is modeled with a logit link.

The exponential, density dependent and autoregressive models have an option to model immigration separately from the growth/decline processes, which can also include a log-linear relationship with site variables. The number of individuals immigrating is estimated as a separate number ($\iota$), which for instance for the exponential model would take the form of

$$N_{i,t} \sim\mathrm{Pois}\left( {N_{i,t-1} e}^{r}+\iota\right)$$

Finally, the third component of the model incorporates detection probability by simulating the observation process. This is where the count data ($X_{i,t}$) comes in:

$$X_{i,t} \sim Bin\left( N_{i,t},p_{i,t} \right)$$

where $p$ is the detection probability. Spatial and temporal variability in the detection probability can be included as a function of $q$ number of observation-level variables ($z_{q,i,j,t}$) with a logit model, such as

$$\ln\left( \frac{p_{i,j,t}}{1-p_{i,j,t}} \right)=\beta_{0}^{p}+\beta_{1}^{p}z_{1,i,j,t}+\beta_{2}^{p}z_{2,i,j,t}\ldots+\beta_{1}^{p}z_{q,i,j,t}$$

Hence, abundance can vary between primary sampling periods $t$ and between sites $i$, whereas detection probability is affected by variables also associated with the exact survey $j$. Maximum likelihood estimates (MLEs) are obtained for all parameters to estimate the model, using … To evaluate competing models, with different variables or dynamics, we can use Akaike’s Information Criterion (AIC) to assess performance and parsimony [26,27]. The AIC calculation uses the maximum log-likelihood ($\log\left( \mathcal{L}\left( \hat{\theta}\left| y \right. \right) \right)$) and the number of estimable parameters ($K$) to provide an information criterion. It penalizes for incorporating too many parameters in a model, thus preserving parsimony:

$$\mathrm{AIC}=-2\log\left( \mathcal{L}\left( \hat{\theta}\left| y \right. \right) \right)+2K$$

The value of the AIC in itself is not useful, it should be used specifically as a comparison measure. We compare the AIC of all candidate models and select the models with the lowest AIC, as it indicates the minimum of the “estimate of the expected, relative distance between the fitted model and the unknown true mechanism that generated the observed data” [27].

To facilitate the interpretation of ‘raw’ AIC values and the differences between models, the Akaike weights for a set of $R$ models can be calculated as:

$$w_{i}=\frac{e^{-0.5 \Delta_{i}}}{\sum_{i=1}^{R} e^{-0.5 \Delta_{i}}}$$

where $\Delta_{i}$ is the difference in AIC between model $i$ and the model with the minimum AIC. This is divided by the sum of these differences. All Akaike weights together add up to 1. Akaike weights represent the conditional probability that a model is the best model (given that one of the $R$ models must be the best model) [27].

*Abundance estimates*

The model does not directly estimate abundance: it estimates parameters of the prior distribution on $N$ and $p$. Using Bayes Theorem and the parameter estimates for $N$ can provide an estimate of the conditional posterior distribution of $N_{i}$ (conditional on the parameters) [20]. So prior parameters are estimated from the marginal likelihood and these are then “plugged into” the conditional posterior distribution of $N_{i}$. The mean of the conditional posterior distribution is then taken as the annual abundance (also referred to as the “empirical best unbiased predictor”).

*Variance Inflation Factor (VIF) analysis*

VIF analysis is conducted to detect and remove collinearity from a dataset with potentially explanatory variables used in regression analysis. For this analysis, every variable $i$ is regressed against all other variables and the R^2^ is then used in the VIF calculation:

$$VIF=\frac{1}{1-R_{1}^{2}}$$

If VIF = 1, there is no collinearity, for $1<VIF\leq5$ there is moderate collinearity, and at VIF > 5 there is substantial collinearity. There is no clear guidelines which value for VIF to use as a cut-off, but in this study we used VIF $\leq$ 5 as the criterion to keep a variable. In the first iteration, all VIFs for each variable are calculated, and the variable with the highest VIF (if > 5) is removed from the dataset. The VIF analysis is then run again on the remaining variables, taking the same approach to variable removal. The process stops once all VIF are $\leq$ 5.
