# Supplementary material for: Evaluation of NEON Data to Model Spatio-Temporal Tick Dynamics in Florida
Source: Insects. 2019 Sep 27;10(10):321. doi: 10.3390/insects10100321 (PMC6836180; doi:10.3390/insects10100321)
Supplement: Supplementary file 1 [file insects-10-00321-s001.zip › Tick_abundance_SupMat_figures_FINAL.docx]

**Supplementary Figures**

Supplementary Figure S1 Comparison of daily precipitation temperature data from NEON and NOAA data.

Supplementary Figure S2 Comparison of daily relative humidity data from NEON and NOAA data.

Supplementary Figure S3 Comparison of daily mean, minimum and maximum temperature data from NEON and NOAA data.

Supplementary Figure S4 Daily observation level variables, extracted from NEON and NOAA data.


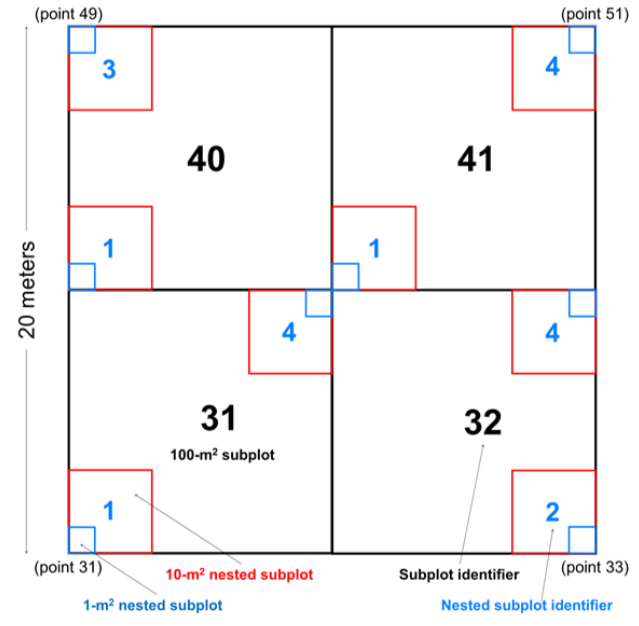


Supplementary Figure S5 Vegetation diversity sampling design [39]
